# Genetic basis of an elite wheat cultivar Guinong 29 with harmonious improvement between multiple diseases resistance and other comprehensive traits

**DOI:** 10.1038/s41598-024-64998-2

**Published:** 2024-06-21

**Authors:** Bei Xiao, Yanmin Qie, Yuli Jin, Ningning Yu, Nina Sun, Wei Liu, Xiaolu Wang, Jiaojiao Wang, Zejun Qian, Ya Zhao, Tangyu Yuan, Linzhi Li, Fengtao Wang, Cheng Liu, Pengtao Ma

**Affiliations:** 1 Yantai Key Laboratory of Characteristic Agricultural Biological Resources Conservation and Germplasm Innovative Utilization, College of Life Sciences, Yantai University, Yantai, 264005 China; 2 Institute of Cereal and Oil Crops, Hebei Academy of Agricultural and Forestry Sciences/Hebei Key Laboratory of Crop Genetic and Breeding, Shijiazhuang, 050035 China; 3 Institute of Grain and Oil Crops, Yantai Academy of Agricultural Sciences, Yantai, 265500 China; 4 Crop Research Institute, Shandong Academy of Agriculture Sciences, Jinan, 250100 China; 5 State Key Laboratory for Biology of Plant Diseases and Insect Pests, Institute of Plant Protection, Chinese Academy of Agricultural Sciences, Beijing, 100193 China

**Keywords:** Wheat, Disease resistance, Stress tolerance, Agronomic and yield traits, Harmonious improvement, Agricultural genetics, Genetic markers, Plant breeding, Plant genetics

## Abstract

Fungal diseases, such as powdery mildew and rusts, significantly affect the quality and yield of wheat. Pyramiding diverse types of resistance genes into cultivars represents the preferred strategy to combat these diseases. Moreover, achieving collaborative improvement between diseases resistance, abiotic stress, quality, and agronomic and yield traits is difficult in genetic breeding. In this study, the wheat cultivar, Guinong 29 (GN29), showed high resistance to powdery mildew and stripe rust at both seedling and adult plant stages, and was susceptible to leaf rust at the seedling stage but slow resistance at the adult-plant stage. Meanwhile, it has elite agronomic and yield traits, indicating promising coordination ability among multiple diseases resistance and other key breeding traits. To determine the genetic basis of these elite traits, GN29 was tested with 113 molecular markers for 98 genes associated with diseases resistance, stress tolerance, quality, and adaptability. The results indicated that two powdery mildew resistance (*Pm*) genes, *Pm2* and *Pm21*, confirmed the outstanding resistance to powdery mildew through genetic analysis, marker detection, genomic in situ hybridization (GISH), non-denaturing fluorescence in situ hybridization (ND-FISH), and homology-based cloning; the stripe rust resistance (*Yr*) gene *Yr26* and leaf rust resistance (*Lr*) genes *Lr1* and *Lr46* conferred the stripe rust and slow leaf rust resistance in GN29, respectively. Meanwhile, GN29 carries dwarfing genes *Rht-B1b* and *Rht-D1a*, vernalization genes *vrn-A1*, *vrn-B1*, *vrn-D1*, and *vrn-B3*, which were consistent with the phenotypic traits in dwarf characteristic and semi-winter property; carries genes *Dreb1* and *Ta-CRT* for stress tolerance to drought, salinity, low temperature, and abscisic acid (ABA), suggesting that GN29 may also have elite stress-tolerance ability; and carries two low-molecular-weight glutenin subunit genes *Glu-B3b* and *Glu-B3bef* which contributed to high baking quality. This study not only elucidated the genetic basis of the elite traits in GN29 but also verified the capability for harmonious improvement in both multiple diseases resistance and other comprehensive traits, offering valuable information for breeding breakthrough-resistant cultivars.

## Introduction

Common wheat (*Triticum aestivum* L.), offering a staple food grain for 35% of the global population and supplying 20% of the caloric intake worldwide, stands as a pivotal agricultural crop in ensuring food supply and security. Multiple diseases seriously affected wheat safety production, including powdery mildew, stripe rust, and leaf rust^2–4^. To combat these diseases, the strategies encompassing cultivation management, chemical prevention, and host resistance have been employed, and the latter is preferred means owe to its high efficiency and environment-friendly characteristics^5,6^. Meanwhile, a systematic evaluation of multiple diseases resistance in those released wheat cultivars at the molecular and genetic levels is also important for their rational distribution in production and application in breeding^7^.

Among wheat diseases, powdery mildew caused by the biotrophic fungus *Blumeria graminis* f. sp. *tritici* (*Bgt*), stripe rust caused by *Puccinia striiformis* f. sp. *tritici* (*Pst*) and leaf rust caused by the *Puccinia triticina* Eriks. are the most widespread and damaging fungal diseases threatening wheat production worldwide^2–4^. To date, more than 100 formally designated *Pm* genes/alleles at 64 loci (*Pm1*-*Pm69*, *Pm8* = *Pm17*, *Pm18* = *Pm1c*, *Pm22* = *Pm1e*, *Pm23* = *Pm4c*, *Pm31* = *Pm21*)^8^, 83 *Yr* genes^9^, and 80 *Lr* genes^10^ have been identified and characterized. These resistance genes were mainly derived from hexaploid common wheat or its ancestral and alien species, such as *T. urartu*, einkorn wheat, emmer wheat, *Aegilops tauschii*, durum wheat, *Ae. squarrosa*, *Ae. speltoides*, *Ae. longissima*, *Ae. ovata*, *Dasypyrum villosum*, *T. urartu*, *T. turgidum* var*. dicoccoides*, *T. turgidum* var. *dicoccum*, *T. turgidum* var*. durum*, *T. timopheevii*, *T. monococcum*, *Thinopyrum intermedium*, and *Secale cereale* (http://wheat.pw.usda.gov/). Most resistance genes cannot be directly applied in wheat breeding due to their undesirable linkage drags, especially the genes derived from wild relatives of wheat^11^. Even in case of resistance genes without linkage drags, two issues still need to be addressed: (1) The most are race-specific and do not provide durable resistance independently, many known resistance genes have lost or are losing the resistant abilities and pyramiding different resistance genes into one cultivar may alleviate this problem^12^; (2) Excessive expression of the resistance genes, might affect other agronomic, yield, and quality performances, hence, cooperative coexistence between diseases resistance and other comprehensive traits is also critical^13^. Therefore, it is necessary to identify resistance genes without linkage drags and with better cooperative capability between other comprehensive traits in wheat breeding programs and production practice.

Molecular marker detection is regarded as a useful, quick, and easy approach for identifying and transferring the resistance genes, especially the diagnostic markers designed by polymorphic nucleotides within the target genes^6^. If the target genes have not been cloned, closely linked markers can also facilitate the detection of these genes^14^. Recently, many elite genes have been identified and confirmed in a large number of wheat genotypes through marker detection, including *Rht-B1b* and *Rht-D1b*^15^, *Yr15*^16^, *Pm2*^17^, *Pm21*^18^, *Pm12*^18^, *PmV*^18^, *Pm24*^2^, and *Vp1-B*^19,20^. Based on these information, the genetic basis of these genotypes were clarified, and better cooperative models between different genes were revealed.

Guinong 29 (GN29) is a wheat cultivar with collaborative improvement between high resistance to multiple wheat diseases and elite comprehensive performance. To dissect the genetic basis of multiple diseases resistance and other key breeding traits and discuss their cooperative improvement capability, the following aspects were carried out in the present study: (i) evaluate its powdery mildew, stripe rust, and leaf rust resistance at both seedling and adult plant stages; (ii) investigate its agronomic and yield performance at different wheat production regions; and (iii) dissect the genetic basis for the multiple diseases resistance and other key breeding traits using genetic analysis, molecular detection, and/or homology-based cloning.

## Materials and methods

### Plant materials

The wheat cultivar GN29 was developed from a cross between the wheat cultivar Guinong 13 (GN13) and the wheat breeding line Guinong 21 (GN21) using marker-assisted selection (MAS) by Guizhou University and Guizhou Sub-center of the National Wheat Improvement Center and released in 2014. The wheat cultivar Ping’an 9 (PA9) was susceptible to powdery mildew and stripe rust and used as the susceptible parent to cross with GN29 to obtain the F_1_, F_2_, and F_2:3_ populations for genetic and lineage analysis of *Pm* and *Yr* genes in GN29. The wheat cultivar Mingxian 169 (MX169) was susceptible to powdery mildew, stripe rust, and leaf rust and used as a susceptible check in multiple diseases resistance evaluation. Nineteen and fourteen wheat donors carrying known *Pm* genes and *Yr* genes, respectively, served as positive controls in the molecular marker detection and/or homology-based cloning experiments (Supplementary Table S1). Donors of these *Pm* and *Yr* genes were provided by Prof. Hongxing Xu, Henan University, Kaifeng, China and Prof. Caixia Lan, Huazhong Agricultural University, Wuhan, China, respectively.

### Resistance assessment to multiple wheat diseases

To assess the powdery mildew resistance, 31 single-spore-derived *Bgt* isolates with different virulence spectra, provided by Prof. Yilin Zhou, Institute of Plant Protection, Chinese Academy of Agricultural Sciences, and Prof. Hongxing Xu, Henan University, were used to test the seedling reaction patterns of GN29 using MX169 as the susceptible control and nine resistant donors with known *Pm* genes as the resistant controls. Five seeds of each genotype were planted in trays (54 × 28 × 4.2 cm) with 128 cells (3.2 × 3.2 × 4.2 cm). When the seedlings grown to the two-leaf stage, they were inoculated with the fresh conidiospores previously developed on the MX169 seedlings. Seedlings in different trays were inoculated with the 31 *Bgt* isolates separately, and each tray was covered with a glass shroud to avoid cross-infection between different isolates. Several resistance stocks with documented *Pm* genes in production or with high resistance were used as resistant controls and MX169 was used as susceptible control. When the pustules were fully developed on the first leaves of MX169 seedlings, approximately 14–15 days after inoculation, infection types (ITs) for each plant were scored based on a 0–4 scale standard, of which ITs 0–2 were regarded as resistant and ITs 3 and 4 as susceptible^21^. All tests were repeated thrice to ensure data reliability.

At the adult stage, GN29 was inoculated with a mixture of all the *Bgt* isolates used in the seedling stage for three consecutive years (2018 to 2020) at Yantai University, Yantai City, Shandong Province, China (121.39′ E, 37.52′ N). Sowing and inoculation methods were referred to our proven technique system^22^. Disease reaction at the adult stage was scored on a 0–9 scale, of which 0–4 was considered as resistant and 5–9 as susceptible^21^. Each plant was assessed twice.

For stripe rust resistance assessment, three *Pst* isolates, CYR32, CYR33, and CYR34 with different virulence spectra were used to test the seedling reaction patterns of GN29 using MX169 as the susceptible control. Assessments of adult plant stripe rust responses were conducted at Yantai University using a mixture of CYR32, CYR33, and CYR34. The sowing and inoculation methods were referred to the reported procedure^23^. The ITs at the seedling and adult plant stages were both scored based on a scale of 0–9, of which ITs ranging from 0 to 6 were classified as resistant, whereas ITs 7–9 as susceptible^24^.

For the leaf rust resistance assessment, mixed *P. triticina* pathotypes collected in Yantai, China were used to test the seedling and adult-stage reaction patterns of GN29 using MX169 as the susceptible control. The sowing and inoculation methods were referred to the reported procedure^18^. Infection types were scored according to the Stakman scale with moderate modification^25^.

### Assessment of agronomic and yield performance

GN29 were planted at Guiyang city, Guizhou Province (26.57 N, 106.71 E), China, area available for popularization and Langfang City, Hebei Province (39.53 N, 116.72 E), China, in a randomized complete block design with three replicates. The wheat cultivars Guinong 19 (GN19) and Yannong 999 (YN999) were used as controls in Guizhou and Langfang, respectively. Each cultivar was planted as a plot with three rows (length: 1.5 m; distance between rows: 0.25 m) and 30 seeds per row. Three plants in the middle of the two internal rows were sampled to evaluate the plant height (PH), spike numbers per plant (SNPP), spikelet numbers per spike (SNS), sterile spikelet numbers per spike (SSNS), kernel numbers per spike (KNS), and thousand-kernel weight (TKW).

### Genetic analysis, molecular marker detection, and homology-based cloning of the Pm genes

To determine the inheritance of powdery mildew resistance in GN29 at the seedling stage, two *Bgt* isolates E09 (prevalent) and E18 (hypertoxic) were used to inoculate GN29 and PA9 and their F_1_ hybrids, F_2_ population, and F_2:3_ families (30 seeds per F_2:3_ family were selected) at the one-leaf stage. After phenotyping, goodness-of-fit was analyzed using the chi-square (*χ*^2^) test to investigate deviations in the observed phenotypic data of the F_2_ populations and F_2:3_ families from the theoretically expected segregation ratios.

Total genomic DNA was isolated using the cetyltrimethylammonium bromide (CTAB) method from young leaves of the wheat seedlings^27^. To detect the *Pm* genes in GN29, 39 diagnostic/linked markers of 31 known *Pm* genes were used to genotype GN29, PA9, and 19 wheat donors with known *Pm* genes (Supplementary Tables S1 and S2). Polymorphic markers were genotyped in the corresponding F_2:3_ families.

PCR amplification and visualization were performed as described in our lab^27^. PCR amplification was carried out in a 10 μL volume system, including 5 μL 2 × *Taq* Master Mix (Vazyme, China), 1 μL 50 ng/μL template DNA and 0.5 μL 10 μM/μL primers. The PCR amplification condition was set as follows: pre-denaturation at 94 °C for 5 min followed by 36 cycles of 94 °C for 30 s, 50–65 °C (depending on the specific primers) for 40 s, 72 °C for 40–120 s (depending on the target bands), finally, extension at 72 °C for 10 min and preservation at 25 °C. The PCR products were then separated on 8% non-denaturing polyacrylamide gels with a 29:1 ratio of acrylamide to bis-acrylamide or 1.5% agarose gel based on the size of the target bands^28,29^.

After confirming the presence of *Pm* genes in GN29, total RNA from the young leaves of GN29 were extracted using the Spectrum Plant Total RNA kit (Sigma-Aldrich, Shanghai, China) following the manufacturer’s recommendations. The RNA samples were quantified by measuring the absorbance at 260 and 280 nm using a NanoDrop 1000 spectrophotometer (Thermo Scientific, Shanghai, China). High-quality RNA was treated with Promega DNase I for cDNA synthesis using Invitrogen SuperScript II reverse transcriptase, according to the manufacturer’s guidelines. Based on the reports of the cloning of *Pm2*^30^ and *Pm21*^31^, the full length of the homologous sequences of *Pm2* and *Pm21* were isolated. After obtaining the coding sequence (CDS) of *Pm2* and *Pm21*, they were sequenced using Sanger sequencing and compared with those of *Pm2*^30^ and *Pm21*^31^.

### Cytogenetic analysis

Genomic in situ hybridization (GISH) was firstly performed to detect *D. villosum* chromatin in the GN29. Mitotic chromosomes of the root tip cells of GN29 were prepared and observed, as previously described^32^. The genomic DNA of *D*. *villosum* was labeled with fluorescein-12-dUTP as a probe to detect chromosomal fragments for GISH. After hybridization with probes, the chromosomes were counterstained with propidium iodide (PI) and mounted on Vectashield (Roche Co., Burlingame, CA, USA). Signals were examined under an Olympus BX60 epifluorescence microscope (Olympus Co., Tokyo, Japan).

To clearly determine the chromosome composition of GN29, we also performed non-denaturing fluorescence in situ hybridization (ND-FISH) to analyze mitotic chromosomes of the root tip cells. The probes in this study were Oligo-pSc199.2-1 (green) and Oligo-pTa535-1 (red), and they were distributed as 5′ end-labeled with 6-carboxyfluorescein (FAM) and 6-carboxytetramethylrhodamine (TAMRA).

### Molecular marker detection of the Yr and Lr genes

To determine the presence of *Yr* genes in GN29, 17 diagnostic/linked markers for known *Yr* genes were used to test GN29 and PA9, using 14 resistant donors with known *Yr* genes as controls (Supplementary Table S2). If the polymorphic band(s) of one *Yr* gene were detected in GN29 and not PA9, this *Yr* gene would most likely exist in GN29. To confirm this result, the polymorphic markers were also used to genotype the segregated populations of GN29 and PA9.

To detect the presence of *Lr* genes in GN29, a similar but simplified procedure was performed using 12 diagnostic/linked markers of known *Lr* genes by comparing the polymorphic band(s) in GN29 and PA9 but not by genotyping the segregation population (Supplementary Table S2). PCR amplification and product visualization were performed as described above^27–29^.

### Molecular marker detection of other key breeding traits

To determine the presence of other key breeding traits in GN29, 45 diagnostic/linked markers closely linked to wheat adaptability, PH, stress tolerance, and quality were used to test GN29, including seven markers for seven vernalization genes (*Vrn-A1c*, *vrn-A1*, *Vrn-B1*, *vrn-B1*, *Vrn-D1*, *vrn-D1*, *Vrn-B3*, and *vrn-B3*), five markers for five dwarfing genes (*Rht-B1b*, *Rht-B1a*, *Rht-D1a*, *Rht-D1b*, and *Rh8*), two markers for two drought tolerance genes (*Dreb1* and *Ta*-*CRT*), and four markers for seven pre-harvest sprouting resistance genes (*TaAFP*-*Bb*, *TaAFP*-*Ba*, *Vp*-*1Ba*, *Vp*-*1Bb*, *Vp*-*1Bc*, *Vp*-*1Bf* and *TaPHS1*) (Supplementary Table S2). PCR amplification and product visualization were performed as described above^27–29^.

## Results

### Agronomic and yield performance of GN29

**Figure 1:**
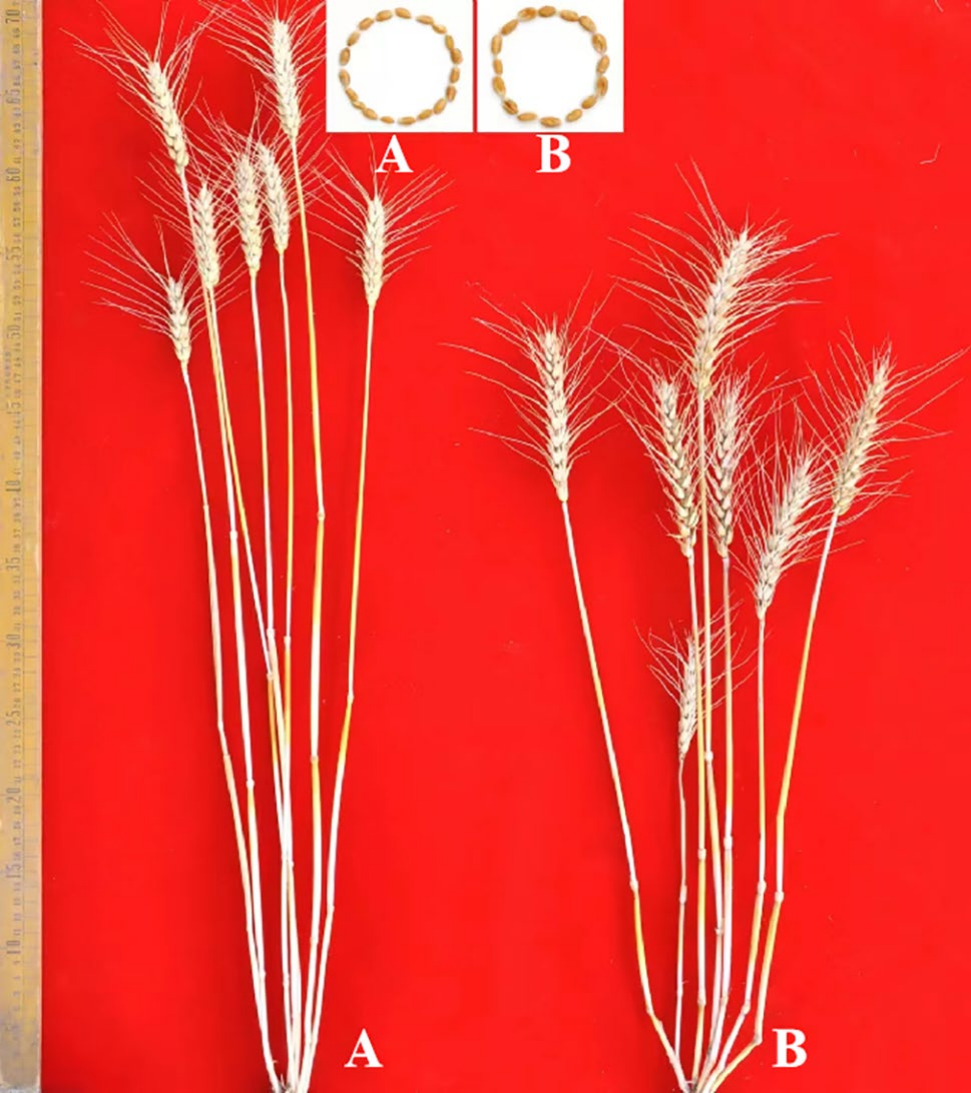
Morphological performance of the wheat cultivars Yannong 999 (**A**) and Guinong 29 (**B**) cultivated in Langfang city, Hebei province, China.

When GN29 was planted in Guizhou Province, it showed comprehensively excellent performance for the investigated traits, including PH, SNPP, SNS, SSNS, KNS, and TKW (Table 1), no obvious disadvantages were detected. Compared with the famous wheat cultivar GN19, GN29 still has significant advantages in terms of SNPP, SSNS, and TKW. To investigate the adaptation of GN29 in other wheat production region, GN29 was also surveyed in Langfang City, Hebei Province. Compared to the performance in Guizhou Province, GN29 significantly increased SNPP but decreased TKW. Significant decrease in the PH was also observed (Fig. 1, Table 1). Although significant changes in agronomic and yield performance occurred in other distinct agroecological area, GN29 still showed better adaptation. Even compared to the famous wheat cultivar YN999, GN29 still showed satisfactory agronomic and yield performance in the Langfang region (Fig. 1, Table 1).

**Table 1:** Agronomic and yield traits of Guinong 29 (GN29) grown in Guizhou and Hebei provinces of China using wheat cultivars Guinong 19 (GN19) and Yannong 999 (YN999) as controls, respectively.

Regions	Genotypes	PH (cm)	SNPP	SNS	SSNS	KNS	TKW (g)
Guiyang, Guizhou Povince, China	GN29	76.5 ± 1.7^a^	7.7 ± 0.6^a^	23.3 ± 0.6^a^	0.3 ± 0.6^a^	63.3 ± 3.5^a^	58.5 ± 0.9^a^
Guiyang, Guizhou Povince, China	GN19	64.4 ± 1.7^b^	6.7 ± 0.6^b^	28.7 ± 2.1^b^	1.7 ± 0.6^b^	66.0 ± 2.0^b^	51.6 ± 1.7^b^
Langfang, Hebei Povince, China	GN29	58.0 ± 2.0^c^	6.7 ± 0.6^b^	22.7 ± 1.2^a^	1.7 ± 0.6^b^	91.0 ± 2.7^c^	39.9 ± 1.5^c^
Langfang, Hebei Povince, China	YN999	70.7 ± 0.6^d^	6.0 ± 1.0^c^	20.3 ± 0.6^c^	1.3 ± 0.6^c^	72.7 ± 2.1^d^	33.8 ± 2.0^d^

Values in the same column followed by the same letter were not significantly different based on the test of *T* test at *P* < 0.05.

PH: plant height; SNPP: spike number per plant; SNS: spikelet number per spike; SSNS: sterile spikelet numbers per spike; KNS: kernel number per spike; TKW: thousand-kernel weight.

### Evaluation and inheritance of the powdery mildew resistance in GN29

When tested with the *Bgt* isolate E09, GN29 showed hypersensitivity on the first leaves and can be regarded as immune with IT 0;, whereas PA9 showed abundant sporulation with > 80% of the first leaves covered with aerial hyphae and hence as highly susceptible with IT 4. The F_1_ plants of GN29 × PA9 showed a similar reaction pattern to E09 as that to GN29 with an IT 0;, suggesting that the *Pm* gene(s) in GN29 displayed dominant inheritance. The F_2_ population fitted the theoretical ratio of 15:1 for the segregation model of the two dominant genes (Table 2).

**Table 2:** Segregation ratios of F_2_ and F_2:3_ generations of Guinong 29 (GN29) and Ping’an 9 (PA9) following inoculation with *Blumeria graminis* f. sp. *tritici* (*Bgt*) isolates E09 and E18 at the seedling stage.

*Bgt* isolates	Cross	Plants observed			Expected ratio	Χ^2^	*P*
		HR	Seg	HS			
E18	GN29 × PA9 F_1_	0	10	0	–	–	–
E18	GN29 × PA9 F_2_	276		105	3:1	1.33	0.24
E18	GN29 × PA9 F_2:3_	95	179	98	1:2:1	0.58	0.75
E09	GN29 × PA9 F_1_	0	10	0	–	–	–
E09	GN29 × PA9 F_2_	143		9	15:1	0.03	0.87
E09	GN29 × PA9 F_2:3_	65	76	8	7:8:1	0.22	0.90

Values of *χ*^2^ for statistical significance at *P* = 0.05 are 3.84 (*df1*) and 5.99 (*df2*); R: Resistant, S: Susceptible, HR: homozygous resistant, Seg: segregating, HS: homozygous susceptible.

Another *Bgt* isolate E18 was also used to inoculate GN29, PA9, and their derived F_1_ plants, F_2_ populations, and F_2:3_ families. Interestingly, a dominant monogenic segregation model was clearly observed using this highly virulent *Bgt* isolate, suggesting that one of the two *Pm* genes in GN29 was defeated by this *Bgt* isolate and the remaining *Pm* gene displayed dominant monogenic inheritance (Table 2).

**Figure 2:**
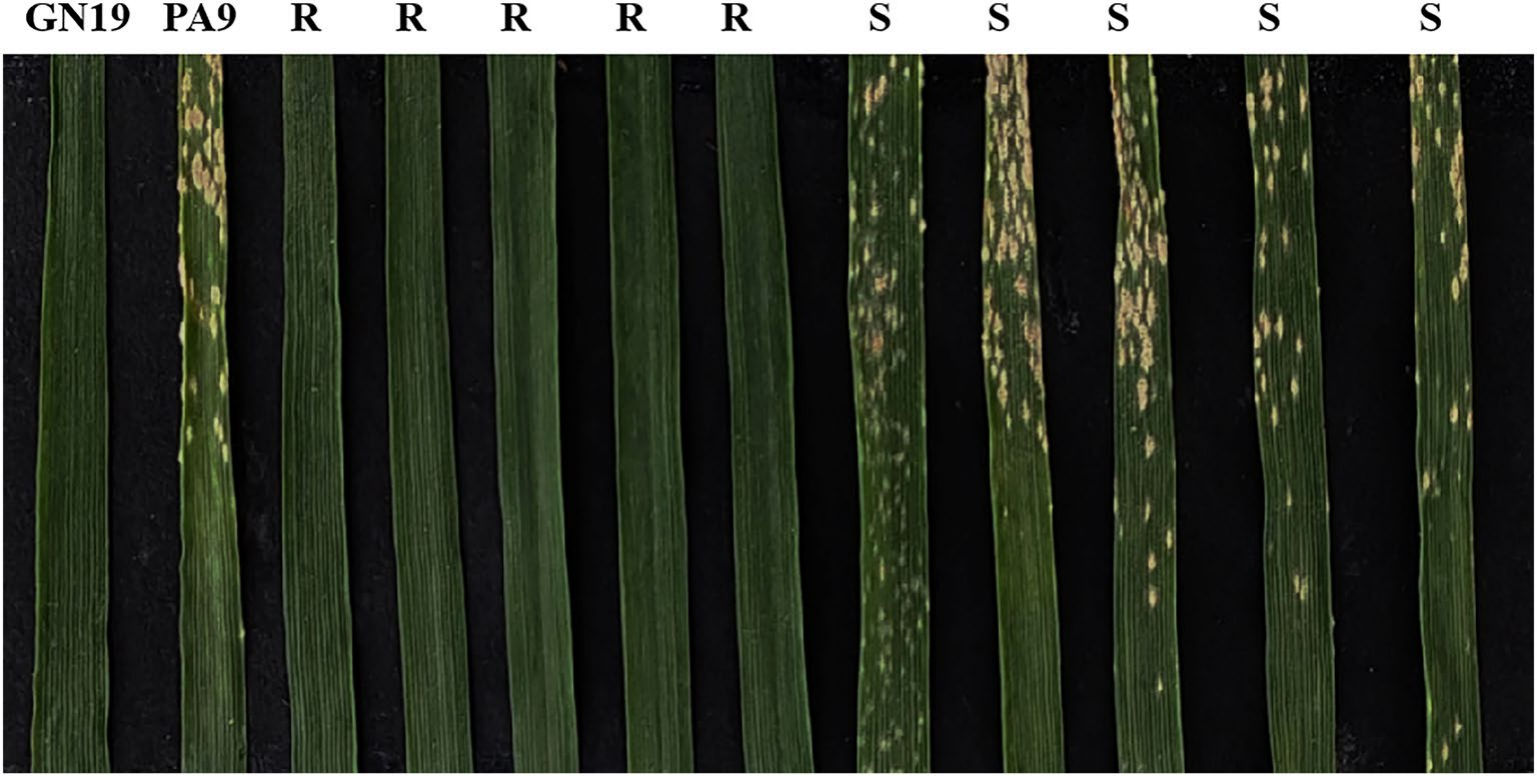
Reaction patterns of Guinong 29 (GN29), Ping’an 9 (PA9) and several resistant and susceptible plants from the F_2_ population of Guinong 29 × Ping’ an 9 inoculated with the *Bgt* isolate E18. R: resistance F_2_ plant; S: susceptible F_2_ plant.

To further assess seedling resistance to powdery mildew in GN29, it was inoculated with 31 *Bgt* isolates using several *Pm* gene donors in production or with a high breeding value as controls. The results showed that GN29 was immune to all the tested *Bgt* isolates (Fig. 2, Table 3). Compared to the other tested *Pm* genes, GN29 had broad spectrum resistance. At the adult stage, GN29 was also immune to the *Bgt* mixture in three consecutive years. Therefore, GN29 showed elite powdery mildew resistance at the whole growth stage.

**Table 3:** Seedling reaction patterns of Guinong 29 and several resistant stocks with documented powdery mildew resistance genes.

*Bgt* isolates	Guinong 29	Ping'an 9	Ulka/8*Cc	Y18	Coker747	Am9/3	66	CH7086	Liangxing 99	ShiCG15-009	YN99102
	*Pm2* + *Pm21*	–	*Pm2*	*Pm21*	*Pm6*	*Pm33*	*Pm37*	*Pm51*	*Pm52*	*PmCG15-009*	*pmYN99102*
E09	0	4	0	4	0	0	0	0	0	0	0
F01	0	4	0	3	4	0	4	4	4	4	4
F02	0	4	0	4	4	0	3	4	0	4	-
F03	0	4	0	4	–	0	0	0	1	3	1
F05	0	4	0	4	4	0	4	4	1	x	–
F06	0	4	0	3	–	0	1	3	4	4	–
F07	0	4	0	4	–	0	0	3	4	4	–
F08	0	4	0	4	4	0	0	0	3	4	–
F09	0	4	0	3	4	0	0	0	0	0;	0
F10	0	4	0	4	4	0	4	4	0	2	4
F11	0	4	0	3	4	0	4	4	0	4	-
F13	0	4	0	4	4	0	0	4	1	2	0
F16	0	4	0	3	4	0	0	0	0	2	4
F17	0	4	0	4	4	0	0	0	0	2	3
F18	0	4	0	3	3	0	0	1	3	3 + 0;	–
F19	0	4	0	4	3	0	0	0	1	3 + 0;	–
F21	0	4	0	4	4	0	4	4	4	4	–
F22	0	4	0	–	–	0	–	–	–	–	–
F23	0	4	0	3	4	0	4	4	4	4	4
F24	0	4	0	4	4	0	4	4	4	x	4
F25	0	4	0	3	4	0	0	3	0	1	3
F28	0	4	0	3	4	0	0	1	0	0;	4
F32	0	4	0	0	–	0	–	–	–	–	–
E05	0	4	0	0	0	0	0	0	–	–	–
E07	0	4	0	4	1	0	1	0	–	–	1
E15	0	4	0	–	–	0	–	–	–	–	4
E17	0	4	0	–	–	0	–	–	–	–	–
E18	0	4	0	4	3	0	0	0	–	–	–
E20	0	4	0	0	0	0	0	0	–	–	–
E21	0	4	0	4	2	0	4	0	–	–	1
E23-1	0	4	0	0	0	0	0	0	–	–	–

Infection types (ITs) were scored according to the 0–4 scale, of which 0, 0; 1 and 2 were considered as resistant, while those with IT 3 and IT 4 were considered as susceptible. ‘–’ represent missing data.

### Molecular detection and homology-based cloning of the Pm genes in GN29

**Figure 3:**
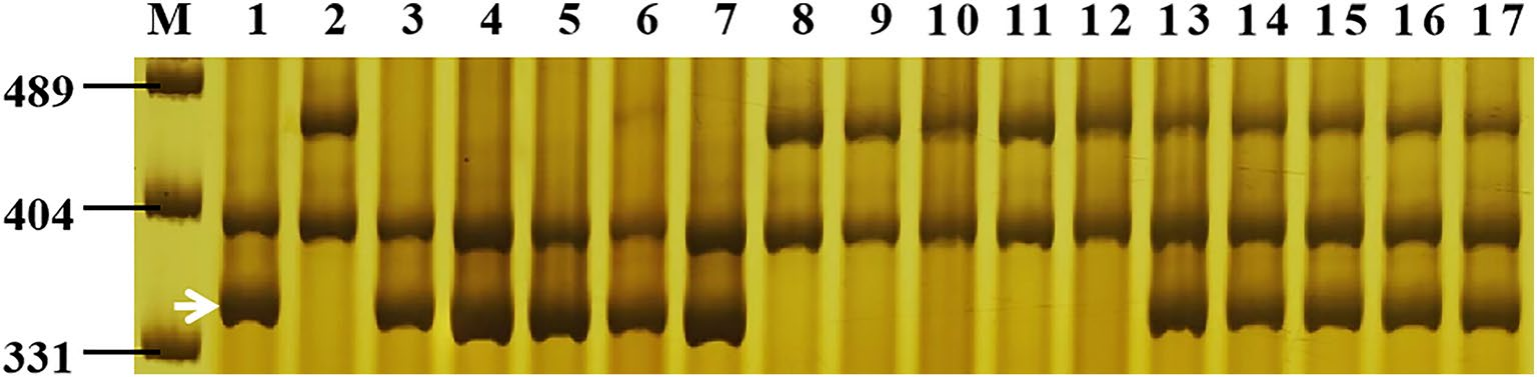
Amplification pattern of the diagnostic marker mbh1 of *Pm21* in genotyping Guinong 29 (GN29), Ping’an 9 (PA9) and random selected F_2:3_ families of GN29 × PA9 at the seedling stage. Lane M, pUC19 *Msp* I; lanes 1–2: parents GN29 and PA9; lanes 3–7: homozygous resistant F_2:3_ families; lanes 8–12, homozygous susceptible F_2:3_ families; lanes 13–17: heterozygous F_2:3_ families. The white arrows indicate the polymorphic bands in GN29.

**Figure 4:**
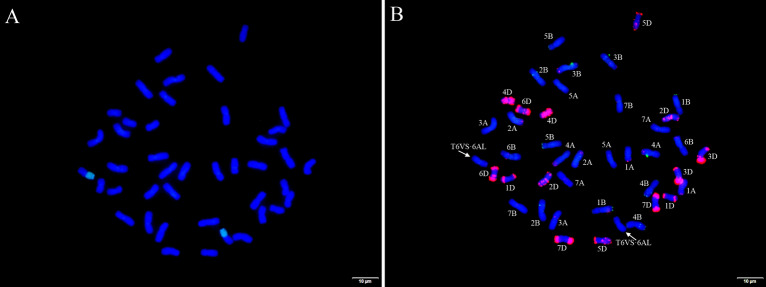
Genomic in situ hybridization (GISH) (**A**) and nondenaturing FISH (ND-FISH) (**B**) analysis of Guinong 29 (GN29). (**A**) GISH analysis on the chromosome constitution in GN29 shows light blue hybridization signals evenly distributed by using *Dasypyrum villosum* genomic DNA as a probe, and the wheat chromosomes were counterstained with 4, 6-diamidino-2-phenylindole (DAPI) (dark blue). (**B**) ND-FISH analysis on the chromosome constitution of Guinong 29 shows no specific hybridization bands on chromosomes of the *D. villosum* in GN29. White arrows note the pair of added 6VS chromosomes.

To identify the *Pm* genes in GN29, 39 diagnostic/linked markers for 31 known *Pm* genes were selected to test GN29 and PA9 **(**Table 4**)**. Only the diagnostic markers *Pm2b-map-3* for *Pm2* and *MBH1* for *Pm21* amplified the targeted bands of *Pm2* and *Pm21*, respectively, in GN29 but not in PA9. To further verify the presence of *Pm2* and *Pm21*, the diagnostic marker *MBH1* of *Pm21* was used to genotype the F_2:3_ families phenotyped by *Bgt* isolate E18. As expected, *MBH1* is co-segregated with the phenotype, suggesting the presence of *Pm21* (Fig. 3). To confirm the existence pattern of *Pm21*, GISH analysis was carried out and showed that GN29 had a pair of alien chromosome arms of *D. villosum* (Fig. 4A)*.* ND-FISH analysis further showed that the alien chromosome arms were 6VS in GN29 (Fig. 4B). The diagnostic marker *Pm2b-map-3* of *Pm2* was also used to genotype the susceptible plants of the F_2_ population phenotyped by the *Bgt* isolate E09, and it co-segregated with the phenotype, suggesting the presence of *Pm2*. To clarify the allelic types of *Pm2* and *Pm21* in GN29, we cloned their homologous sequences. Following sequence alignment, the haplotypes in GN29 were confirmed as *Pm2a* and *Pm21*.

**Table 4:** The presence/absence of different genes in Guinong 29 (GN29), Guinong 13 (GN13) and Guinong 21 (GN21).

Gene	GN29	GN13	GN21	Gene	GN29	GN13	GN21	Gene	GN29	GN13	GN21
Pm1	−	−	−	Lr1	+	-	+	TaAFP-Bb/TaAFP-Ba	+	+	+
Pm2	+	+	−	Lr9	−	−	−	Vp-1Bb/Vp-1Ba/Vp-1Bc	+	-	+
Pm4	−	−	−	Lr10	−	−	−	Vp-1Ba/Vp-1Bf	+	+	+
Pm5	−	−	−	Lr19	−	−	−	TaPHS1	−	−	−
Pm6	−	−	−	Lr20	−	−	−	Vrn-A1c	−	−	−
Pm8	−	−	−	Lr24	−	−	−	vrn-A1	+	+	−
Pm12	−	−	−	Lr26	−	−	−	Vrn-B1	+	+	−
Pm16	−	−	−	Lr34	−	−	−	Vrn-D1	−	−	−
Pm17	−	−	−	Lr37	−	−	−	Vrn-B3	+	+	+
Pm21	+	−	+	Lr46	+	+	+	By8	−	−	−
Pm24	−	−	−	Yr1	−	−	−	Bx14	−	−	−
Pm33	−	−	−	Yr5	−	−	−	Dx5	−	−	−
Pm34	−	−	−	Yr9	−	−	−	Dy10	−	−	−
Pm35	−	−	−	Yr10	−	−	−	Dy12	−	−	−
Pm38	−	−	−	Yr15	−	−	−	Glu-A3a	−	−	−
Pm39	−	−	−	Yr17	−	−	−	Glu-A3b	−	−	−
Pm41	−	−	−	Yr18	-	-	-	Glu-A3ac	−	−	−
Pm42	−	−	−	Yr24	-	-	-	Glu-A3d	−	−	−
Pm45	−	−	−	Yr26	+	-	+	Glu-A3e	−	−	−
Pm47	−	−	−	Yr29	-	-	-	Glu-A3f.	−	−	−
Pm50	−	−	−	Yr30	-	-	-	Glu-A3g	−	−	−
Pm52	−	+	−	Yr41	-	-	-	Glu-B3a	−	−	−
Pm55	−	−	−	Yr67	-	-	-	Glu-B3b	+	+	−
Pm56	−	−	−	YrSP	-	-	-	Glu-B3c	−	−	−
Pm58	−	−	−	Rht-B1b	+	+	+	Glu-B3d	−	−	−
Pm59	−	−	−	Rht-B1a	−	−	−	Glu-B3e	−	−	−
Pm60	−	−	−	Rht-D1b	−	−	−	Glu-B3fg	−	−	−
Pm61	−	−	−	Rht-D1a	+	+	+	Glu-B3g	−	−	−
Pm64	−	−	−	Rht8	+	−	+	Glu-B3h	−	−	−
Pm65	−	−	−	Dreb1	+	+	+	Glu-B3i	−	−	−
Pm69	−	−	−	Ta-CRT	+	+	−	Glu-B3bef	+	+	−

### Evaluation and identification of stripe rust and leaf rust resistance in GN29

When tested with *Pst* isolates CYR32, CYR33, and CYR34 at the seedling stage, GN29 showed high resistance with IT 1. When tested with a mixture of these three *Pst* isolates at the adult plant stage, GN29 also showed high resistance ranging from IT1-2. For the leaf rust resistance assessment, GN29 was highly susceptible to the mixed *Pt* races; however, it showed slow reactance at the adult plant stage, suggesting that the slow leaf rust resistance gene may be involved in GN29.

**Figure 5:**
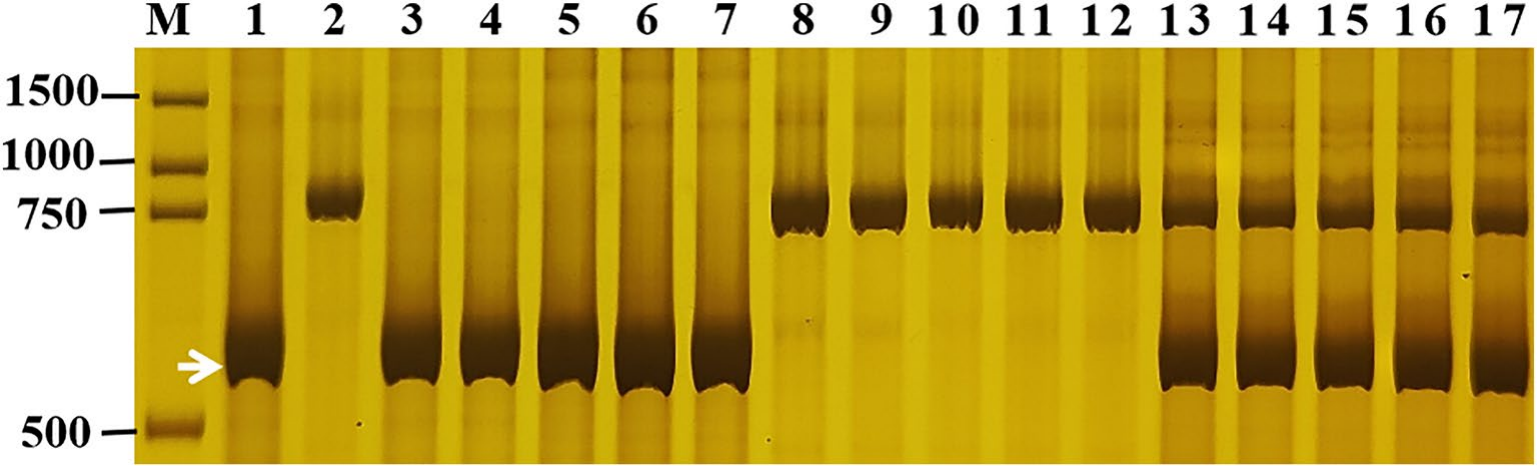
Amplification pattern of WE173 linked to *Yr26* in genotyping Guinong 29 (GN29), Ping’an 9 (PA9) and random selected F_2:3_ families of GN29 × PA9 at the seedling stage. Lane M, pUC19 *Msp* I; lanes 1–2: parents GN29 and PA9; lanes 3–7: homozygous resistant F_2:3_ families; lanes 8–12, homozygous susceptible F_2:3_ families; lanes 13–17: heterozygous F_2:3_ families. The white arrows indicate the polymorphic bands in GN29.

To investigate the *Yr* gene(s) in GN29, 17 diagnostic/linked molecular markers for 14 known *Yr* genes were used to test GN29 **(**Table 4**)**. The results showed that only the *Yr26*-linked marker *WE173* could amplify the target band, suggesting that GN29 was most likely to carry *Yr26*. To further verify the presence of *Yr26*, *WE173* was used to genotype the F_2:3_ families phenotyped by isolate CRY34. The result showed that *WE173* was co-segregated with phenotypes, further confirming the presence of *Yr26* (Fig. 5). A simple and similar procedure was also carried out to detect the *Lr* gene(s) in GN29 by analyzing the polymorphic band(s) in GN29 but not by genotyping the segregation population, suggesting that *Lr1* and *Lr46* may exist in GN29 (Supplementary Table S2).

### Molecular identification of drought tolerance and preharvest sprouting resistance in GN29

To investigate the drought tolerance and preharvest sprouting resistance genes in GN29, six markers linked to two main drought tolerance genes and three markers linked to three main preharvest sprouting resistance genes were used to detect GN29 **(**Table 4**)**. The results indicated that markers P18, P20, P21, P22, and P25 associated with the drought tolerance gene *Dreb1*, and DF/DR linked to *Ta-CRT*, successfully amplified target bands, suggesting that GN29 was most likely to carry *Dreb1* and *Ta-CRT*, implying the potential drought tolerance of GN29 (Supplementary Table S2). However, marker detection results also indicated that GN29 had none of the three main preharvest sprouting resistance genes, *TaAFP-Bb/TaAFP-Ba*, *Vp-1Bb/Vp-1Ba/Vp-1Bc*, and *Vp-1Ba/Vp-1Bf*, implying potential risks in pre-harvest sprouting (Supplementary Table S2).

### Molecular identification of vernalization and dwarfing genes in GN29

To investigate the vernalization and dwarfing genes in GN29, nine markers linked to eight vernalization genes and five markers linked to five dwarfing genes were used to detect GN29. The results indicated that the markers BF-WR1 linked to *Rht-B1b*, DF-MR2 linked to *Rht-D1a* and gwm261 linked to *Rht8* could amplify the target bands, suggesting that GN29 most likely carries the dwarfing genes *Rht-B1b*, *Rht-D1a*, and *Rht8* (Table 4, Supplementary Table S2). The PH of GN29 planted in Guizhou Province did not correlate with the presence of *Rht-B1b*, *Rht-D1a*, and *Rht8*; however, the PH in Hebei Province was mostly affected by their presence. For the detection of vernalization genes, GN29 had four of the seven tested vernalization genes *vrn-A1*, *Vrn-B1*, *vrn-D1*, and *Vrn-B3*, hence, GN29 could be considered a semi-winter cultivar. The better adaptation in Guizhou and Hebei Provinces may be related to this factor.

### Molecular identification of quality-related genes in GN29

To investigate the quality-related genes in GN29, 22 markers linked to 22 glutenin subunit genes were used to detect GN29. The result indicated that the markers SB2 linked to *Glu-B3b* and SB10 linked to *Glu-B3bef* could amplify the target bands, suggesting that GN29 was most likely to carry two low-molecular-weight glutenin subunit genes, *Glu-B3b* and *Glu-B3bef*, which contribute to the malleability of the dough and food processing quality (Table 4, Supplementary Table S2). We noticed that the elite high-molecular-weight glutenin subunit genes *Dx5* and *Dx10* were absent in GN29, which may affect the gluten strength of GN29 (Table 4, Supplementary Table S2).

## Discussion

Wheat breeding involves the integration of elite traits from diverse donors into a unified genetic background. In this process, harmonious improvement between different traits is critical, particularly between multiple diseases resistance and other comprehensive traits^13^. For instance, it is difficult to pyramid large mumble of elite traits between multiple diseases resistance and stress tolerance, adaptation, quality, and high yield and better express all of them in a harmonious pattern^33^. In this study, we identified an elite wheat cultivar, GN29, which achieved harmonious improvement between multiple diseases resistance and a number of other elite traits.

GN29 is an elite wheat cultivar with resistance to multiple important diseases. For powdery mildew resistance, it pyramided two elite *Pm* genes, *Pm2* and *Pm21*, both of which have high breeding value. *Pm2* was initially identified in the wheat landrace Ulka from the former Soviet Union in 1953^34^. Over the following 70 years, *Pm2* was adequately used in wheat production and showed exceptional performance in fighting wheat powdery mildew^34^. To date, *Pm2* still shows high and broad-spectrum resistance in some genetic backgrounds, such as the wheat cultivars/breeding lines 10 V-2, YingBo 700 and KM2939, and the landrace Niaomai^27,36–38^. A similar situation occurs for *Pm21*, which is currently the most effective *Pm* gene and has been used in at least 20 cultivars^39^. Given the high value of *Pm2* and *Pm21*, extending their operation lifespan during wheat production is imperative. Gene pyramiding is a promising strategy for developing durable resistance. Fortunately, *Pm2* and *Pm21* are pyramided in GN29 and achieve better collaboration, which is expected to be interdependent and can build durable resistance against continuous *Bgt* variations. In addition to powdery mildew resistance, GN29 also pyramided one *Yr* gene, *Yr26*, and two different kinds of *Lr* genes, *Lr1* and *Lr46*. Among them, *Yr26* is an elite resistance gene with high- and broad-spectrum resistance to stripe rust throughout the whole growth stage and has been used in production for many years^40^; *Lr1* is a frequently used resistance gene with leaf rust resistance throughout the whole growth stage^41^; *Lr46* is a resistance gene with slow resistance to leaf rust, and this locus is also resistant to stripe rust (*Yr29*)^42^, powdery mildew (*Pm39*)^43^, and stem rust (*Sr58*)^44^ as a multiple resistant locus. Therefore, it is rare to pyramid so many resistance genes into a single cultivar, and meanwhile realize better collaboration.

Beyond the five different resistance genes, GN29 also pyramided two genes conferring drought tolerance, which implies its potential drought resistance ability and may be suitable for extension and application in arid and water-scarce regions. The *Dreb* gene is an important gene involved in abiotic stress tolerance in wheat production, including tolerance to drought, salinity, low temperature, and ABA^45^. The combination of *Dreb* and the five resistance genes further suggests that GN29 may possess a cooperative ability to improve both biotic and abiotic stresses.

GN29 also pyramided two low-molecular-weight glutenin subunit genes, *Glu-B3b* and *Glu-B3bef*, but no high-molecular-weight glutenin subunit genes, particularly the elite *Dx5*, were detected in GN29*.* Therefore, GN29 is regarded as a low-gluten cultivar and valuable for producing low-gluten flour, which is mainly used to make biscuits.

Given the extensive pyramiding of genes related to biotic and abiotic stress, as well as quality in GN29, an important question arises regarding the impact of these genes on agricultural and yield performance. From the agricultural and yield analyses, we found that GN29 maintained elite agricultural and yield performance and no obvious defects were observed. Meanwhile, GN29 can be considered a semi-winter cultivar based on the detection of vernalization genes^46–48^, suggesting that it is suitable for extension and application in southwestern wheat production regions, such as Guizhou, Yunnan, and Sichuang provinces of China, and in the south-central region of the northern winter wheat region. Agricultural and yield performances in distinct regions also indicated that GN29 has better adaptability, whether in Guizhou Province in the south or Hebei Province in the north. In addition, differences in plant height were obvious in different regions. Although three dwarfing genes were identified in GN29, they could not be adequately displayed in the Guizhou Province where GN29 has been selected and popularized. In Hebei Province, the three dwarfing genes were fully displayed, which may be related to different ecoclimatic conditions.

## Conclusion

The wheat cultivar GN29 showed promising coordination between multiple diseases resistance and other key breeding traits. To determine the genetic foundation of these elite traits, GN29 was tested with 113 molecular markers for 98 target genes associated with diseases resistance, stress tolerance, quality, and adaptability. Several key genes were confirmed using genetic analysis, marker detection, and/or homology-based cloning. This study not only dissect the genetic basis of GN29 but also verified the harmonious improvement ability across multiple diseases resistance and other key breeding traits, which can provide elite gene resources and references for gene pyramiding during breeding practices.

### Supplementary Information

Supplementary Figure S1.

Supplementary Figure S2.

Supplementary Table S1.

Supplementary Table S2.

## Data Availability

All data generated or analyzed during this study are included in this published article. We declare that the plant materials used for our study were provided by Chinese Crop Germplasm Resource Bank (Beijing, China) as seed materials and could be also purchased by commercial channel. We did not use endangered plant species for the experiments. Experimental research and field studies on plants (either cultivated or wild), including the collection of plant material, complied with relevant institutional, national, and international guidelines and legislation.
