# Supplementary figures and images for: Genetic basis of an elite wheat cultivar Guinong 29 with harmonious improvement between multiple diseases resistance and other comprehensive traits
Source: Sci Rep. 2024 Jun 21;14:14336. doi: 10.1038/s41598-024-64998-2 (PMC11192888; doi:10.1038/s41598-024-64998-2)

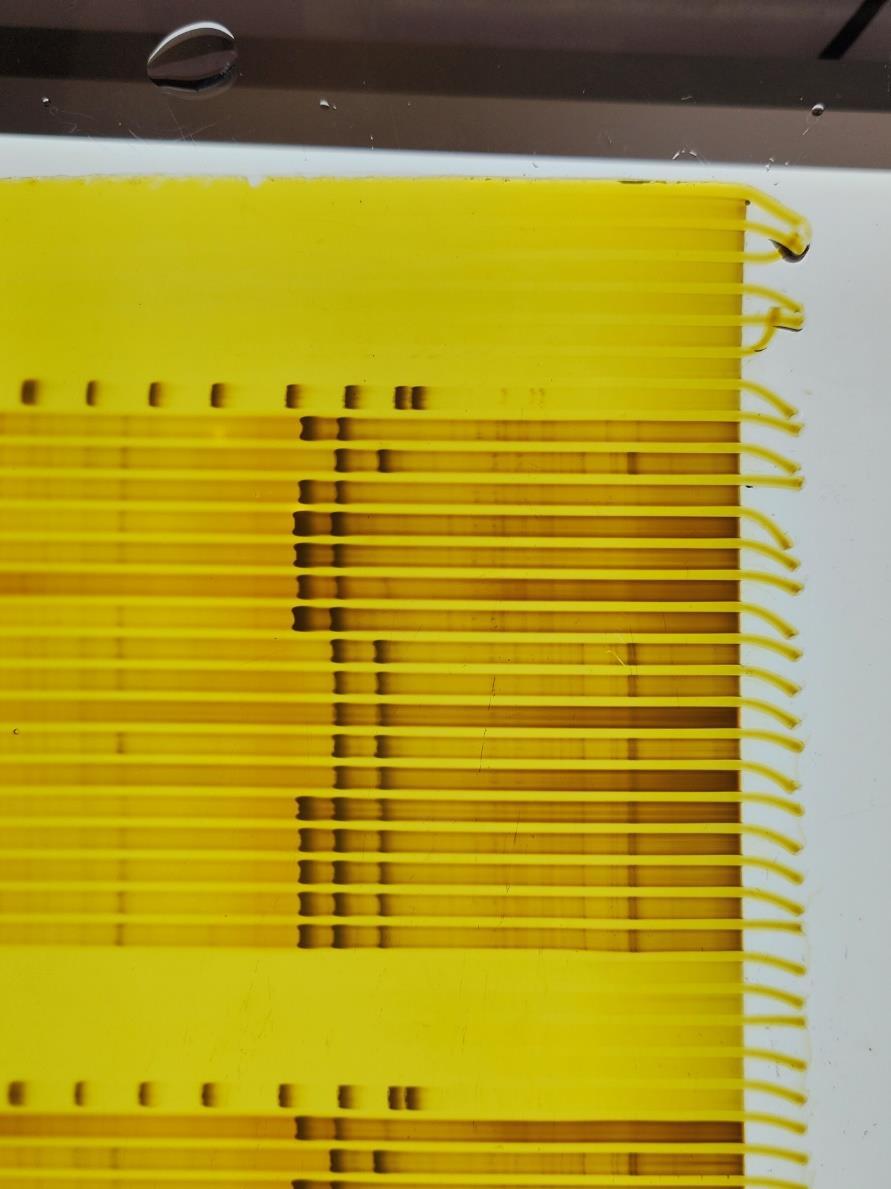

Supplement: Supplementary file 1 — Supplementary Figure S1. [file 41598_2024_64998_MOESM1_ESM.jpg]

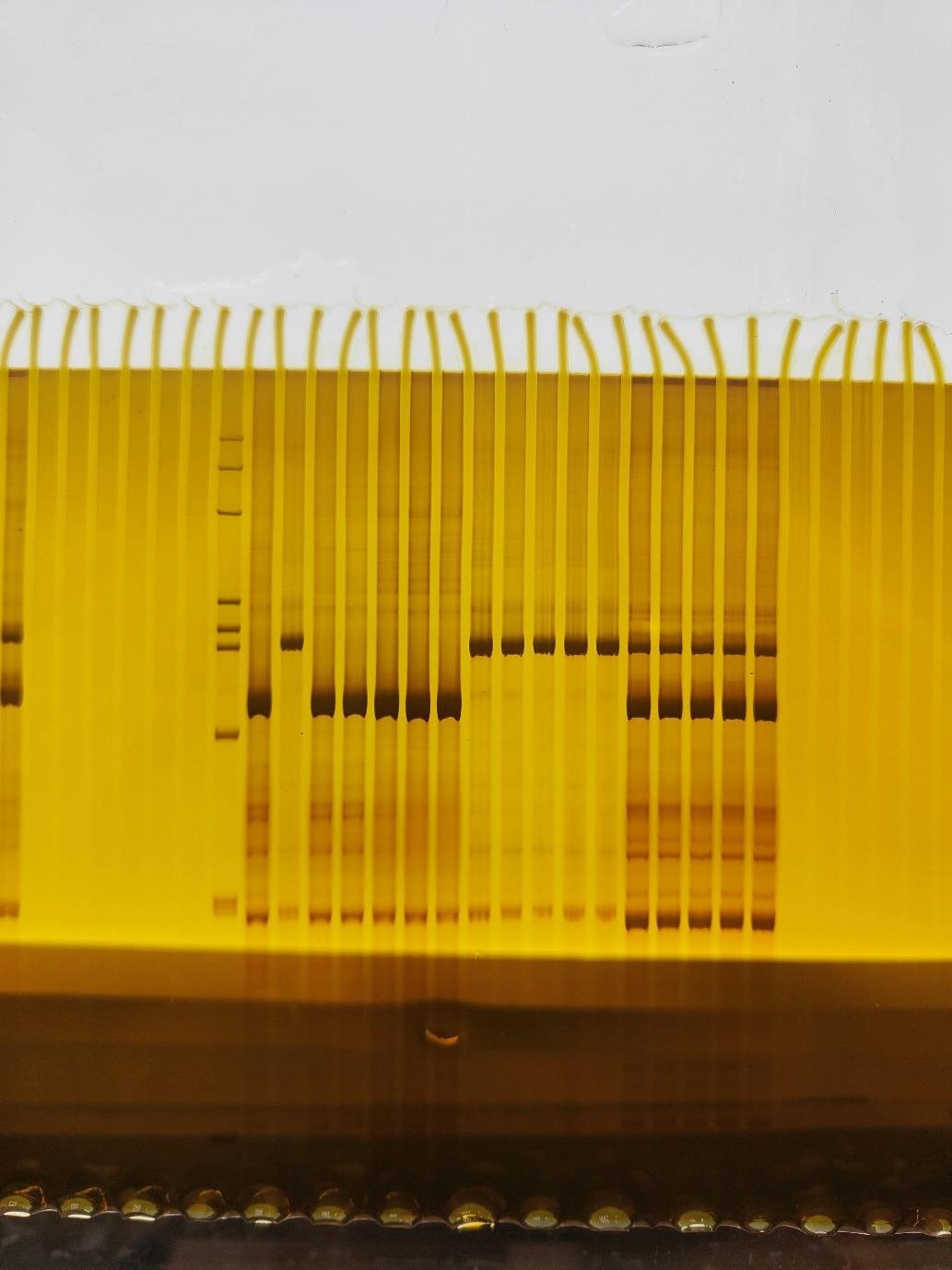

Supplement: Supplementary file 2 — Supplementary Figure S2. [file 41598_2024_64998_MOESM2_ESM.jpg]
